# Analyses of point mutation repair and allelic heterogeneity generated by CRISPR/Cas9 and single-stranded DNA oligonucleotides

**DOI:** 10.1038/srep32681

**Published:** 2016-09-09

**Authors:** Pawel Bialk, Brett Sansbury, Natalia Rivera-Torres, Kevin Bloh, Dula Man, Eric B. Kmiec

**Affiliations:** 1 Gene Editing Institute, Helen F. Graham Cancer Center and Research Institute, Newark, Delaware, United States of America; 2 Department of Medical Laboratory Science, College of Health Sciences, University of Delaware, Newark, Delaware, United States of America; 3 Nemours Center for Childhood Cancer Research, Alfred I. duPont Hospital for Children, Wilmington, Delaware, United States of America; 4 Department of Chemistry, Delaware State University, Dover, Delaware, United States of America

## Abstract

The repair of a point mutation can be facilitated by combined activity of a single-stranded oligonucleotide and a CRISPR/Cas9 system. While the mechanism of action of combinatorial gene editing remains to be elucidated, the regulatory circuitry of nucleotide exchange executed by oligonucleotides alone has been largely defined. The presence of the appropriate CRISPR/Cas9 system leads to an enhancement in the frequency of gene editing directed by single-stranded DNA oligonucleotides. While CRISPR/Cas9 executes double-stranded DNA cleavage efficiently, closure of the broken chromosomes is dynamic, as varying degrees of heterogeneity of the cleavage products appear to accompany the emergence of the corrected base pair. We provide a detailed analysis of allelic variance at and surrounding the target site. In one particular case, we report sequence alteration directed by a distinct member of the same gene family. Our data suggests that single-stranded DNA molecules may influence DNA junction heterogeneity created by CRISPR/Cas9.

Single-stranded DNA oligonucleotides (ssODNs) can be used to direct single base exchange in organisms ranging from yeast^1^,^2^,^3^,^4^,^5^, to plants^6^,^7^,^8^,^9^ to mammalian cells^10^,^11^,^12^,^13^. We have been studying the mechanism and regulation of single agent gene editing elucidating the regulatory pathways^12^,^14^,^15^. We have also utilized ssODNs in combination with programmable nucleases and RNA –guided engineered nucleases (RGENs) to execute point mutation repair in mammalian cells^16^,^17^,^18^. In this approach, the oligonucleotide is designed so that it invades the target duplex with a 3′ to 5′ polarity, consistent with traditional reaction mechanics of homologous pairing^19^,^20^,^21^,^22^. A critical reaction intermediate, known as a D-loop, is thus created and the resolution of this structure is the basis of single agent gene editing. This strategy directs base exchange to proceed through mismatch repair or through ssODN incorporation into a growing replication fork. In addition, we and others established that cells undergoing DNA replication, passing through S phase, are more amenable to gene editing activity^23^,^24^,^25^,^26^,^27^,^28^, consistent with this overall mechanism of action. For gene editing strategies, however, where the goal is to insert single-stranded DNA ultimately to execute gene replacement, the polarity with which the oligonucleotide interacts with the site is less stringent because it does not need to invade the duplex fully^29^,^30^,^31^,^32^. This pathway of gene editing utilizes the process of Homology Directed Repair (HDR).

In this study, we examine how successful single base gene editing correlates with CRISPR/Cas9 cleavage activity in a reaction where the objective is to correct a single base mutation in a well-established model gene editing system where both genotypic and phenotypic readout have been validated^17^. We sought to define the relationship between point mutation repair and DNA cleavage activity since the product of CRISPR/Cas9 activity is often a heterogeneous population of chromosomal ends created through DNA resection promoted by non-homologous end joining (NHEJ). DNA cleavage assays have often been used as a benchmark to measure the level of successful gene editing in mammalian cells, especially with regard to genetic knockout. While such correlations are likely to be valid for studies where the goal is to disable the gene, our experience led us to believe that gene editing of a single base mutation could be accompanied by variable DNA changes at the target site. Here, we examine the outcome of the combined activity of single-stranded oligonucleotides and a specifically designed CRISPR/Cas9 system at a specific target site with a particular emphasis on the evaluation of allelic mutagenesis.

## Results and Discussion

**Figure 1:**
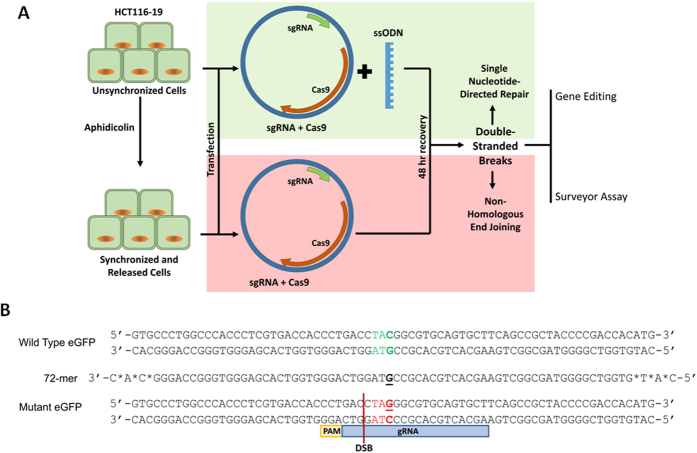
(**A**) Experimental workflow. HCT116-19 cells are either unsynchronized or synchronized and released, then transfected with a CRISPR/Cas9 expression vector (pX330) with or without ssODN, then after 48 hours analyzed for gene editing activity and Surveyor endonuclease digestion. **(B)** Gene editing model system and ssODNs. The wild-type and mutated eGFP gene segments with the target codon located in the center of the sequences are displayed in green and red, respectively. The nucleotide targeted for exchange is emphasized in bold and underlined. The gRNA and protospacer adjacent motif (PAM) shown indicate the CRISPR/Cas9 target site and the location of the resulting double-stranded break (DSB). The phosphorothioate modified, end protected 72-mer which is used to target the mutated eGFP gene is shown.

The repair of a single point mutation by gene editing can be evaluated using a well-established reporter gene system consisting of a single copy of a mutant eGFP gene integrated into HCT116 cells^11^,^15^,^30^. Repair of this mutation is executed by the combined action of a specifically designed ssODN, 72 bases in length, and the appropriate CRISPR/Cas9 system^17^. The cells can be targeted as either an unsynchronized population or synchronized and released population, as shown in Fig. 1A. The reaction is initialized through strand invasion by the ssODN and subsequent alignment in homologous register with the target site (here, the mutant eGFP gene) except for a single base pair mismatch located in the center^33^. Figure 1B illustrates the alignment of the ssODN with the appropriate strand of the mutant eGFP gene. Here, we evaluated gene editing activity by the combined action of the ssODN, and a CRISPR/Cas9 system previously designed for the same target^11^,^17^ and concurrently measured CRISPR/Cas9 cleavage activity using the well-known Surveyor endonuclease assay^34^. Since this is a reporter gene, off-target effects are minimized, yet we include the ten top predicted off target sites in supplementary Table S1.

**Figure 2:**
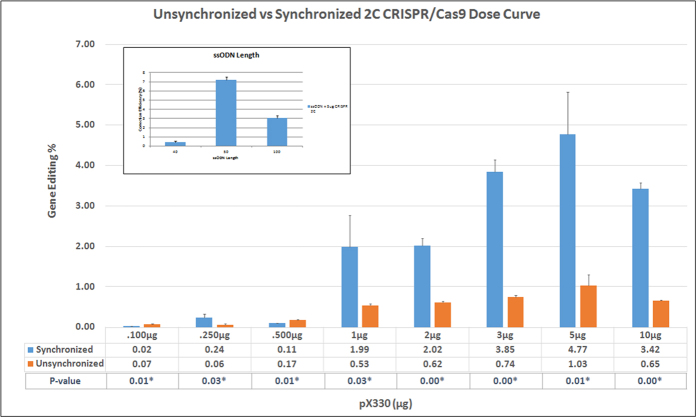
Gene editing dose curve using in synchronized and unsynchronized cells. Synchronized (blue) and unsynchronized (orange) HCT116-19 cells were electroporated with 0.1–10.0 μg of pX330 and 1.35 μg of 72mer. After a 48-hour recovery period, gene editing activity was measured using a Guava EasyCyte 5HT Flow Cytometer. Gene editing is displayed as correction efficiency (%), determined by the number of viable eGFP positive cells divided by the total number of viable cells in the population. Each treatment was performed in triplicate and standard error is illustrated with accompanying bars. Statistical analysis was performed using two-sample unequal variance students T-test distribution to compare the value of correction efficiency between synchronized and un-synchronized cells when treated with CRISPR/Cas9. *p < 0.05. Inset. Correction efficiency using varying lengths of ssODN at equimolar concentrations with 5 ug of pX330, 48 hours after electroporation.

The 72 base ssODN (72-mer) and the CRISPR/Cas9 expression construct were introduced by electroporation and wild type eGFP expression measured 48 hours later by FACS. The amount of ssODN was fixed and the level of CRISPR/Cas9 expression construct was increased in a stepwise fashion. Figure 2 illustrates the level of gene editing activity obtained from a population of cells that have either been treated in an unsynchronized state or synchronized and released prior to the addition of the ssODN and CRISPR/Cas9 (expressed from plasmid, pX330). Gene editing activity is dose-dependent, exhibiting higher levels when synchronized and released cells are targeted as compared to cells targeted in the unsynchronized state. Consistent with previous findings, synchronization and release increases the percentage of cells transiting S phase at the time of DNA addition, a reaction condition that has been shown to increase the frequency of gene editing^30^,^31^,^32^,^35^,^36^. Activity is predictably reduced when higher levels of the expression constructs are added due, in all likelihood, to extensive DNA cleavage activity and cellular toxicity^16^,^17^. We’ve also confirmed that the optimal length of a single-stranded DNA molecules for gene editing falls within the range of 50–100^30^,^31^ bases, in the eGFP system (see inset in Fig. 2).

**Figure 3:**
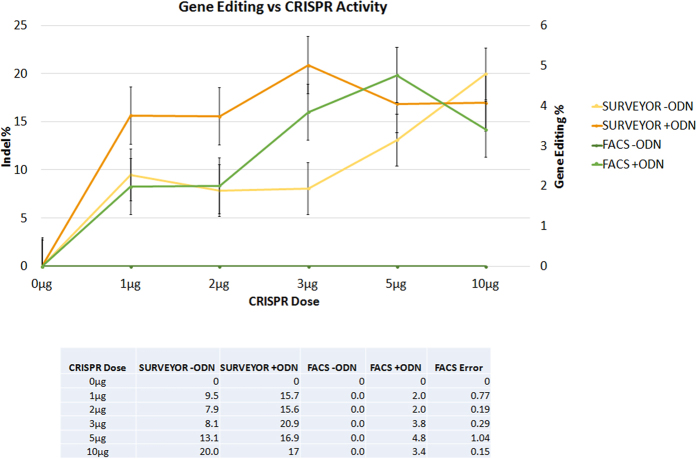
Correlation between CRISPR/Cas9 cleavage and gene editing activity. Synchronized and released HCT116-19 cells were electroporated with 0.0–10.0 μg of pX330 and with (+ODN) or without (−ODN) 1.35 μg of 72-mer. CRISPR/Cas9 cleavage activity measured by Surveyor endonuclease assay (orange and yellow) as well as gene editing (dark green and light green) activity measured by FACS are shown. Standard error is represented by the bars on the each data point.

Next, DNA cleavage activity, generated through the action of the CRISPR/Cas9 system, was determined by the Surveyor endonuclease at dosages that support significant levels of gene editing. The data are presented in Fig. 3 and supplementary Figure S1. The Surveyor assay successfully detected CRISPR/Cas9 activity in the absence of the ssODN; a predictable rise in activity was observed as a function of the amount of expression vector present in the reaction. When cleavage activity is measured in complete reaction mixtures, *i. e.* in the presence of the oligonucleotide, Surveyor endonuclease activity is increased (orange line vs yellow line), particularly at the two doses where gene editing activity is near maximal. While this may appear to be counterintuitive, the Surveyor endonuclease assay has a preference for DNA duplexes with small but definitive indels. DNA duplexes bear small insertions, deletions or single base changes which are more appropriate as substrates for cleavage^34^. This may suggest a significant degree of heterogeneity at the target site in conjunction with point mutation repair or perhaps independent of it. A similar observation was made by Schumann *et al*. using a T7 Endonuclease I (T7E1) recognition assay to measure CRISPR/Cas9 cleavage activity^37^. In that system, however, the objective was different; to insert a segment of DNA.

**Figure 4:**
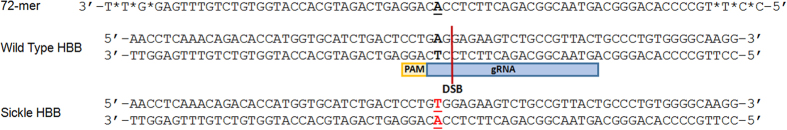
Experimental strategy for targeting the human HBB gene. The wild-type and Sickle Cell HBB gene sequences are shown with the single nucleotide mutation characteristic of Sickle Cell Disease emphasized in red. The gRNA and protospacer adjacent motif (PAM) shown indicate the CRISPR/Cas9 target site and the location of the resulting double-stranded break (DSB). The phosphorothioate modified, end protected 72-mer is used to create a mismatch at the mutation site indicated in bold.

The correlation between the extent of single base gene editing and the degree of heterogeneity detected by the Surveyor assay prompted us to examine the DNA sequence both at and surrounding the target site, as a function of point mutation repair. A testable hypothesis is that point mutation repair could be accompanied by various degrees of onsite mutagenesis, resulting in a population of heterogeneous re-ligated DNA ends, the resection products. In order for our observations and data to have more relevance, we decided to do a detailed analysis of single base repair, as it may relate to onsite mutagenesis, on a native gene target. The beta globin gene in K562 cells is a well-established and well-known target for gene editing particularly with regard to Sickle Cell Disease^38^,^39^,^40^. A mutation in the sixth codon of the beta globin gene is responsible for the onset of Sickle Cell Disease and, as such, a reversal of that point mutation through gene editing would have immediate translational applications. We utilized K562 cells which harbor 2–3 copies of the normal beta globin gene^41^,^42^, bearing a wild type nucleotide (A), at position 16 in the coding region (GAG), as displayed in Fig. 4. We employed the same strategy used for eGFP gene editing; a CRISPR/Cas9 system and the appropriate single-stranded oligonucleotide to convert the A to a T nucleotide.

**Figure 5:**
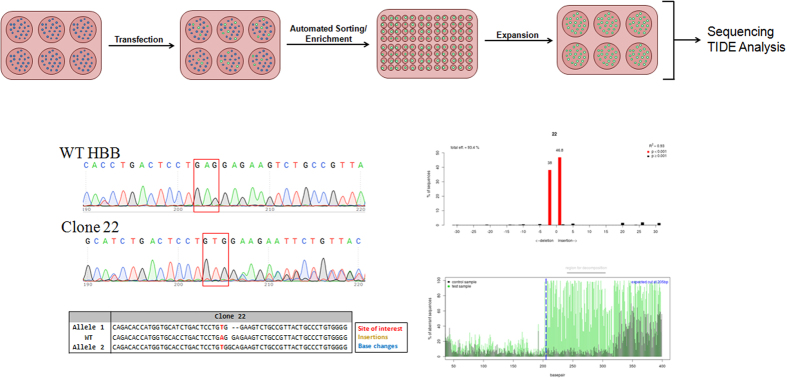
HBB gene editing experimental flow. The K562 cells were transfected with the CRISPR/Cas9 and ssODN followed by a 72 hour incubation period. Targeted cells exhibiting eGFP expression were sorted using a FACSAria II flow cytometer as single cells into individual wells for clonal expansion. DNA was then isolated and the HBB gene was amplified and subjected to Sanger sequencing and TIDE analyses to investigate the gene editing activity around the target site of the clonally expanded populations.

A CRISPR/Cas9 complex that induces DNA cleavage at the indicated site (Fig. 4) was chosen because it had the lowest potential for off-target activity yet would still induce a DNA break site closest to the target nucleotide in the beta globin gene. We also depict the 72-mer oligonucleotide that has been designed specifically to create a mismatch within the adenosine nucleotide (A, in bold) within the wild-type beta-globin (HBB) gene. Resolution of this point mutation through gene editing results in the creation of a T at the mismatch site and subsequently a T:A base pair at the target site (illustrated in Red). The CRISPR/Cas9 targeting system is harbored in an expression vector which also contains a wild-type eGFP gene so that K562 cells successfully transfected are discernible by FACS. The overall experimental flow, as illustrated in Fig. 5, was designed with the ultimate goal of full-scale analyses of sequence alterations. The cells exhibiting eGFP expression were sorted using a FACS Aria II and placed into individual wells for single cell clonal expansion. Cells were allowed to expand for approximately 3–5 weeks at which time DNA was extracted from the clonal expansions and prepared for sequence analysis. Figure 5 (lower panels) displays the results of the Sanger sequencing of 1 individual/expanded clonal population (Clone 22) as well as Sanger sequencing trace files analyzed by the software program, Tracking of Indels by decomposition (TIDE)^43^. The TIDE program facilitates the examination of individual allelic sequences within complex Sanger sequencing data, which often displays a multiplicity of nucleotide peaks at or surrounding the position of DSB repair. An algorithm that isolates individual sub-sequences followed by an alignment mechanism relative to the control parental sequence to determine the indel structure of individual alleles is utilized. In addition, TIDE affords the opportunity for qualitative analysis of clonally expanded populations, analyses that can provide unique insight into the cleavage profile of each allele in individual targeted cells. As an example of our analytical strategy, we display data from Clone 22 which harbors homozygosity for the biallelic A to T conversion, as indicated by the nucleotides presented in red. We know that while point mutation repair has occurred, each allele contains a different indel; the TIDE indel distribution displays a 2 base deletion (allele 1) and a 1 base insertion (allele 2).

**Figure 6:**
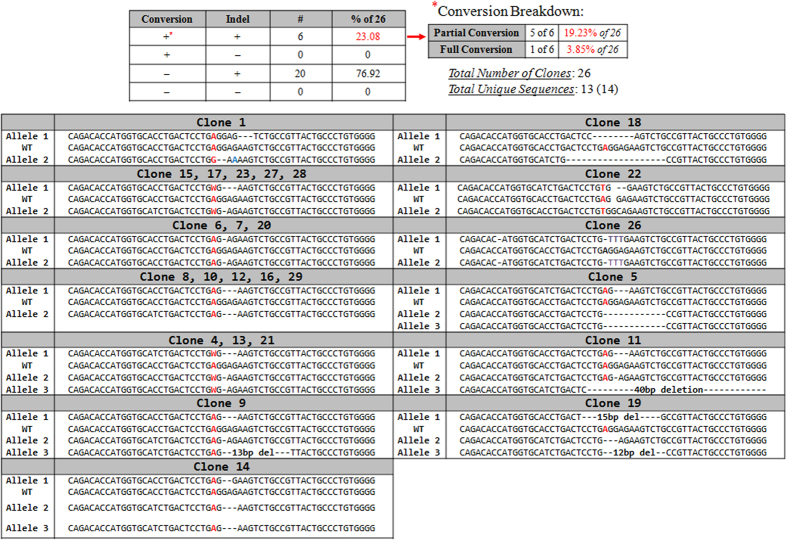
Clonal analysis of the targeted HBB gene. Individual clones, expanded from targeted populations, were analyzed for indel formation at the site surrounding the target nucleotide. Allelic analysis was carried out using Sanger sequencing and TIDES. The small box at the top of the figure illustrates the four potential outcomes of the targeting events: precise point conversion alone, conversion accompanied by indel formation, indel formation alone, and no genetic change. The symbol (^#^) indicates the number of clones which aligned with this description adjacent to the calculation of percent of that population displaying that genetic content within the 26 clones analyzed. The conversion (*) breakdown refers to how many events bearing partial (at least one allele converted) and full (all alleles converted) conversion were seen in 23.08% of the clones that within themselves showed point mutation repair. The allelic breakdown is presented in several groups, where appropriate, demonstrating that a number of clones exhibited an identical indel formation pattern. Due to the variation of chromosomal content within K562 cells, either 2 or 3 alleles, as indicated, were mapped using the strategy. For convenience, we provide the sequence of a wild-type allele, in the center of the panel surrounded by the sequences of gene 2 or 3 targeted alleles from the indicated clone.

Allelic analysis was performed on 26 individual clones and among them 23.08% (6) contained at least one allele with the repaired nucleotide. Of the 6 clones having some degree of conversion, 5 of the 6 had at least one allele corrected (partial conversion); the other one had both alleles corrected (full conversion). Figure 6 and Supplemental Figure S2 depict data from the four possible genotypic outcomes of the gene editing reaction and detailed clonal analyses. These include clones that have the precisely repaired point mutation (conversion) alone, conversion accompanied by indel formation, indel formation alone or clones with no genetic change. The data show that 100% of the 26 clones contained indels while 23% contained specific point mutation repair on at least one allele. Through Sanger sequencing and TIDE analyses, we found that a majority of the indels harbor between 1 and 3 base deletions. Additionally, Sanger sequencing and TIDE analyses reveal several distinct recombination patterns around the double-stranded break site. Of the 26 clones analyzed, only 14 distinct sequences were found surrounding the break site, and in several cases, relatively large groups of these clones were identical genetically. For example, 5 of the 26 clones displayed a 3 base pair deletion on one allele and a one base deletion on the other. A similar pattern was also observed with 5 additional clones, all of which had a 3 base pair deletion on both alleles. This interesting result is significant in that these 2 distinct groups comprise 38.46% of all the clones analyzed. Likewise, another 2 distinct groups emerge from our study. One group, making up 11.54% of analyzed clones, displayed a 1bp deletion directly downstream of the cleavage site in both alleles. The second group, which comprised another 11.54% of all analyzed clones, contained three HBB alleles, as determined by proportions of sub-sequence prevalence in the TIDE readout; one allele contained a 3 bp deletion, and the other 2 alleles both contained a 1bp deletion downstream of the cut site. Overall, this amounts to 61.54% of all of the individually isolated clonal populations exhibiting 1 of 4 indel patterns.

Taken together, these results indicate that single point mutation repair, catalyzed by the combination of a single-stranded oligonucleotide and a specific CRISPR/Cas9, can generate a population of cells with corrected alleles and a heterogeneous mix of sequence alterations at or surrounding the target site. These results could also suggest that onsite mutagenesis in gene editing systems where point mutation repair is the objective, could impact the translation of the technology into a more relevant clinical setting, perhaps for Sickle Cell Disease. The importance of these data will impact gene editing studies with a central focus of identifying and analyzing converted cells but not evaluating the population of cells *not* displaying the desired phenotype; the so-called uncorrected population. Deep sequencing of offsite mutagenesis remains a central focus of most analytical approaches for gene editing, but our data suggest that onsite mutagenesis may be significant.

**Figure 7:**
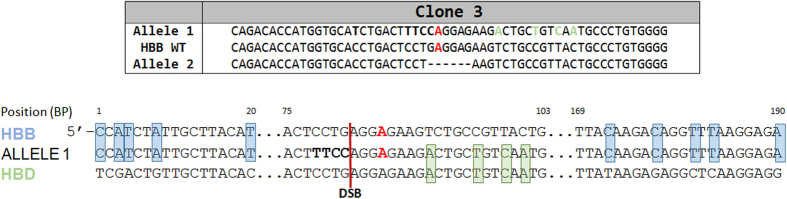
Template-based repair of the beta globin gene (HBB) directed by the delta globin gene (HBD). The same experimental strategy as outlined in the legend of Fig. 5 was employed in the analysis of Clone 3. Allele 1 is displayed alongside the analogous sequences of WT HBB and WT HBD. Points of homology exclusively between HBB and the sequence of Allele 1 are shown in blue, as seen distally upstream and downstream of the cleavage site. Points of homology exclusively between HBD and Allele 1 are shown in green.

Finally, an interesting result emerged from the analysis of one clone (Clone 3). The allelic sequence variation of both alleles is displayed in Fig. 7 with an accompanying sequence diagram. We observe a curious pattern of DNA nucleotides adjacent to the double-stranded break site; footprints of the human delta globin gene. In other words, one allele of Clone 3 appears to be a chimera of the HBB gene and the hemoglobin delta gene (HBD). The blue highlights indicate single nucleotide signatures of HBB while the green highlighted bases indicate signatory nucleotides of HBD. Interestingly, allele 2 harbors simple deletion and in this case neither allele was corrected at the target base. While this outcome may be unique, and is only seen in one the 26 clones analyzed, it does suggest the genes with similar sequences (related family members) such as HBB and HBD could be involved in template repair of double strand DNA breaks independent of exogenously added donor DNA. We are currently pursuing this line of investigation to either establish or minimize the involvement of this type of molecular repair event.

## Methods and Materials

### Cell Line and Culture Conditions

HCT116 cells and K562 cells were acquired from ATCC (American Type Cell Culture, Manassas, VA). The HCT116-19 was created by integrating a pEGFP-N3 vector (Clontech, Palo Alto, CA) containing a mutated eGFP gene^11^. The mutated eGFP gene has a nonsense mutation at position +67 resulting in a nonfunctional eGFP protein. For these experiments, HCT116 -19 cells were cultured in McCoy’s 5A Modified medium (Thermo Scientific, Pittsburgh, PA) supplemented with 10% fetal bovine serum, 2 mM L-Glutamine, and 1% Penicillin/Streptomycin. Cells were maintained at 37 °C and 5% CO_2_. K562 cells were cultured in Iscove’s Modified Dulbecco’s Medium (ATCC, Manassas, VA), supplemented with 10% fetal calf serum and 1% Penicillin/Streptomycin. Cells were maintained at 37 °C and 5% CO_2_. The eGFP targeting custom designed 72-mer oligonucleotide was synthesized by IDT (Integrated DNA Technologies, Coralville, IA). The HBB custom 72-mer single-stranded oligonucleotide used (5′-CCTTGCCCCACAGGGCAGTAACGGCAGACTTCTCCACAGGAGTCAGATGCACCATGGTGTCTGTTTGAGGTT-3′), was obtained from TriLink Biotechnologies, San Diego, CA.

### CRISPR Design and Construction

The guide RNA and CRISPR/Cas9 for the eGFP system used for gene editing in this system was described previously^17^. CRISPR/Cas9 were constructed using standard cloning methods following the latest oligo annealing and backbone cloning protocol with single-step digestion-ligation^44^. The eGFP target gRNA was cloned into pX330 backbone vector (Addgene plasmid 42230), a human codon-optimized SpCas9 and chimeric guide RNA expression plasmid. The HBB gene sequence was entered into the Zhang Lab’s online generator (http://crispr.mit.edu/) and the appropriate CRISPR guide sequence which binds in close proximity to target (+2bp downstream of the sickle mutation site) was chosen. The gRNA was cloned into the pX458 backbone vector (Addgene plasmid 48138), a human codon optimized pSpCas9 and chimeric guide RNA expression plasmid with a 2A-eGFP. pX330 and pX458 were purchased through Addgene (https://www.addgene.org). Following construction, clones were verified by DNA sequencing by Genewiz Incorporated (South Plainfield, NJ).

### Experimental Strategy

For experiments utilizing synchronized cells, HCT116-19 cells and K562 cells were seeded at 2.5 × 10^6^ cells in a 100 mm dish and synchronized with 6 μM aphidicolin for 24 hours prior to targeting. Cells were released for 4 hours prior to transfection by washing with PBS (−/−) and adding complete growth media. Synchronized and unsynchronized HCT116-19 cells were simultaneously transfected at a concentration of 5 × 10^5^ cells/100 μl in 4 mm gap cuvette (BioExpress, Kaysville, UT). 0.6 μM of single-stranded oligonucleotide and/or the appropriate dose of pX330 constructs were electroporated (250 V, LV, 13 ms pulse length, 2 pulses, 1 s interval) using a Bio-Rad Gene Pulser XCell^TM^ Electroporation System (Bio-Rad Laboratories, Hercules, CA). Cells were then recovered in 6-well plates with complete growth media at 37 °C for 24–48 hours prior to analysis. For K562 cell transfections, synchronized and unsynchronized cells were seeded in6-well plates at a density of 5 × 10^5^ cells/well in 1.5 mL complete media. Transfection complexes of 2.5 μg pX458 containing the targeting gRNA and 0.6 μM 72-mer ssODN were formed at room temperature in 500 uL Opti-MEM according to the optimized LTX protocol (Thermo-Fisher, Waltham, MA) and added drop-wise to K562 cells, followed by a 72 hour incubation. After incubation, individual cells were sorted into each well of a 96-well plate with a FACSAria II flow cytometer (BD Biosciences, San Jose, CA). Clones were expanded into larger plates as the individual clones reached confluence, with DNA isolation occurring when cells reached confluence in a 6-well plate (~1 × 10^6^ cells/mL).

### Analysis of Gene Edited Cells

HCT116-19 cell fluorescence (eGFP^+^) was measured by a Guava EasyCyte 5HT Flow Cytometer (Millipore, Temecula, CA). Cells were harvested by trypsinization, washed once with 1x PBS (−/−) and resuspended in buffer (0.5% BSA, 2 mM EDTA, 2 μg/mL Propidium Iodide (PI) in PBS −/−). Propidium iodide was used to measure cell viability as such, viable cells stain negative for PI (uptake). Correction efficiency was calculated as the percentage of the total live eGFP positive cells over the total live cells in each sample. Cellular gDNA was isolated from pellets of 1–2 × 10^6^ K562 cells using the Qiagen DNEasy Blood and Tissue Kit (Cat. ID 69506, Valencia, CA). PCR was performed using Phusion High-Fidelity PCR Master Mix with HF Buffer (Thermo-Scientific, Waltham, MA) on isolated gDNA, with amplification parameters optimized for an amplicon size of 345 bp (FWD Primer: 5′- TCCTAAGCCAGTGCCAGAAGAG -3′, REV Primer: 5′- CTATTGGTCTCCTTAAACCT -3′, obtained from Integrated DNA Technologies, Coralville, IA). Amplicon size was verified on 1% agarose gel, and PCR products were verified by DNA sequencing by Eton Bio Incorporated (Union, NJ).

Individual K562 cell clones were analyzed by the software program, **T**racking of **I**ndels by **De**composition (TIDE), to determine the individual sub-sequences within the multi-peaked breakdown product after CRISPR/Cas9 activity (Netherlands Cancer Institute, https://tide.nki.nl/). The TIDE analyses provide a visual of the sequence decomposition, the indel patterns of the clone, as well as relative ratios of each clonal indel pattern, serving as an intermediate step in determining each allelic profile. By utilizing the indel patterns and their relative ratios provided by TIDE, the control trace sequence and a clonal trace sequence were manually aligned, allowing for the visualization of the indel patterns of each allele of a particular clone.

### SURVEYOR Analysis of CRISPR/Cas9 Cleavage Activity

HCT116-19 cells were electroporated at a concentration of 5 × 10^5^ cells/100 μl in 4 mm gap cuvette (BioExpress, Kaysville, UT) with pX330 or pX330 and 1.35 μg of the 72 mer ssODN. Cells were then recovered in 6-well plates with complete growth media at 37 °C for 72 hours. DNA was isolated using the Blood and Tissue DNeasy kit (Qiagen, Hilden, Germany). The Surveyor assay was performed on 605 bp amplicons that were created using forward primer, 5′CTGGACGGCGACGTAAACGGC and reverse primer, 5′ACCATGTGATCGCGCTTCTCG. PCR samples were purified using the QIAquick PCR purification kit (Qiagen, Hilden, Germany). 200 ng of each PCR product was mixed with 200 ng of PCR product from the untreated sample and subjected to a heteroduplex formation: 95 °C for 10 minutes, 95 °C to 85 °C with a ramp rate of −2 °C/s, 85 °C for 1 minute to 75 °C at −0.1 °C/s, 75 °C for 1 minute to 65 °C at −0.1 °C/s, 65 °C for 1 minute to 55 °C at −0.1 °C/s, 55 °C for 1 minute to 45 °C at −0.1 °C/s, 45 °C for 1 minute to 35 °C to 25 °C at −0.1 °C/s, 25 °C for 1 minute. After duplex formation products were treated with SURVEYOR Nuclease S and SURVEYOR Enhancer S (IDT Technologies) for 30 minutes at 42 °C, gel electrophoresed and stained with SYBR Safe DNA stain (Life Technologies). Gels were imaged with a Gel Doc EZ Imager (Bio-Rad) and densitometry was performed by measuring the area under the curves of each band, using the Image Lab software (Bio-Rad). Calculations were based on the following formulas:


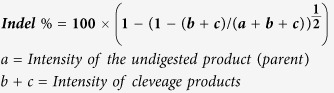


## Additional Information

**How to cite this article**: Bialk, P. *et al*. Analyses of point mutation repair and allelic heterogeneity generated by CRISPR/Cas9 and single-stranded DNA oligonucleotides. *Sci. Rep.* **6**, 32681; doi: 10.1038/srep32681 (2016).

## Supplementary Material

Supplementary Information
