# Supplementary material for: Analyses of point mutation repair and allelic heterogeneity generated by CRISPR/Cas9 and single-stranded DNA oligonucleotides
Source: Sci Rep. 2016 Sep 9;6:32681. doi: 10.1038/srep32681 (PMC5016854; doi:10.1038/srep32681)
Supplement: Supplementary Information [file srep32681-s1.pdf]

Analyses of point mutation repair and allelic heterogeneity generated by CRISPR/Cas9 and single stranded DNA oligonucleotides.

Pawel Bialk[1], Brett Sansbury[2], Natalia Rivera-Torres[3], Kevin Bloh[1,4] Eric B. Kmiec[1,2]*

[1] Gene Editing Institute, Helen F. Graham Cancer Center and Research Institute, Newark, Delaware, United States of America

[2] Department of Chemistry, Delaware State University, Dover, Delaware, United States of America

[3] Department of Medical Laboratory Science, College of Health Sciences, University of Delaware, Newark, Delaware, United States of America

[4] Nemours Center for Childhood Cancer Research, Alfred I. duPont Hospital for Children, Wilmington, Delaware, United States of America

*** Corresponding Author**

***eric.b.kmiec@christianacare.org**

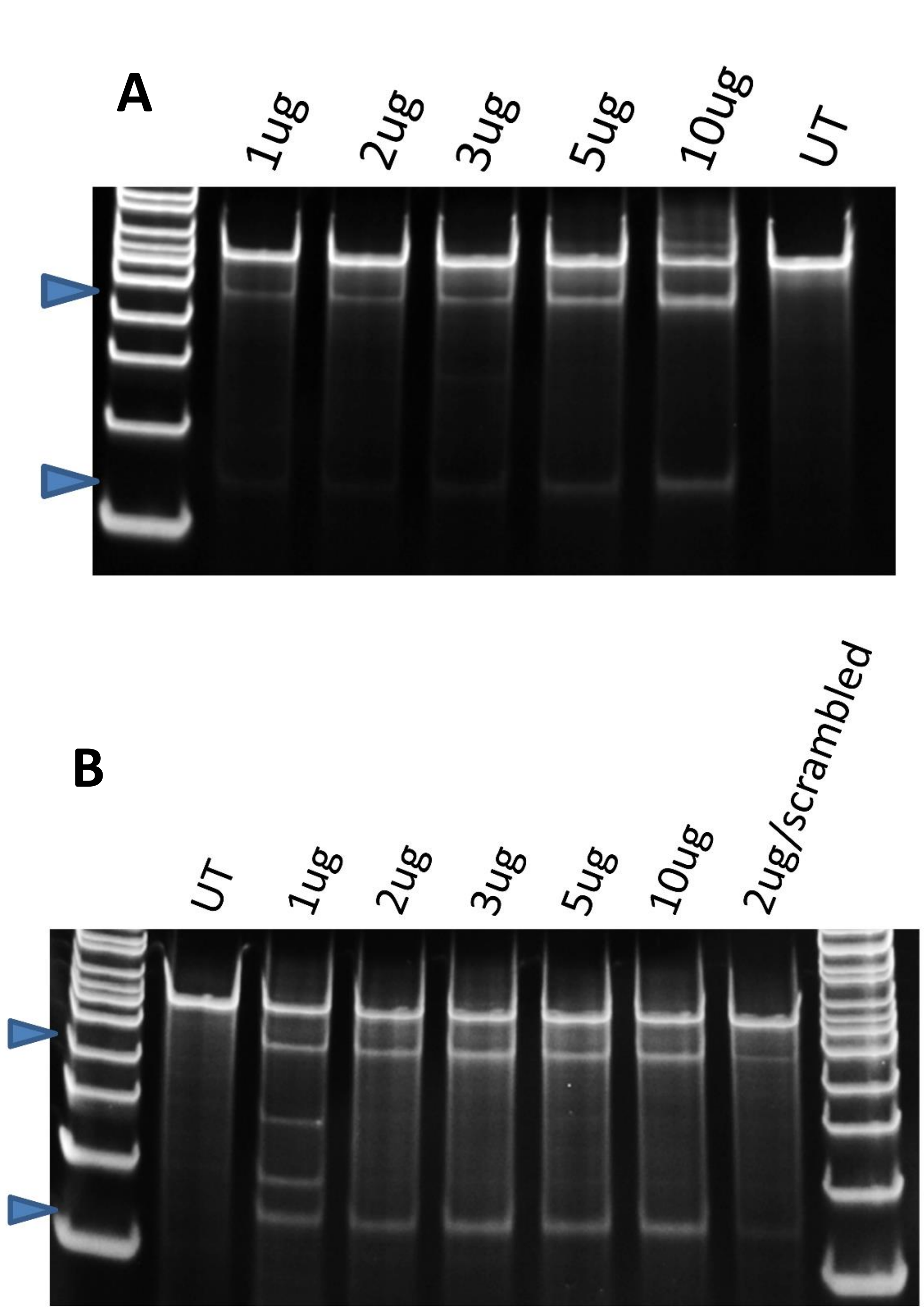

Supplementary S1. Surveyor Analysis. (A) Surveyor analysis of synchronized HCT116-19 cells that were transfected with increasing concentrations of pX330 containing the eGFP targeting gRNA. Cleavage products are indicated by the small arrows. (B) Surveyor analysis of synchronized HCT116-19 cells that were transfected with increasing concentrations of pX330 containing the eGFP targeting gRNA and 1.35ug of the 72-mer ssODN or 2ug of pX330 with a 72-mer scrambled oligo. Cleavage products are indicated by the small arrows.

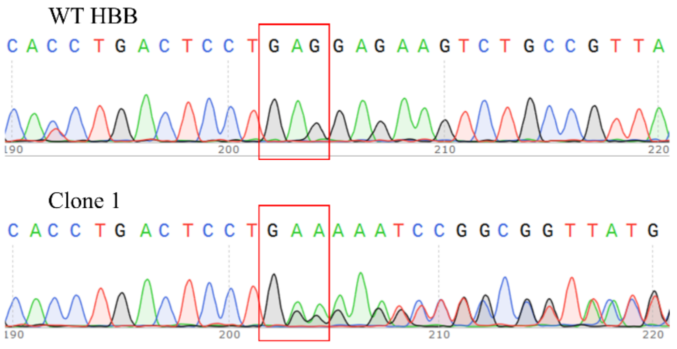

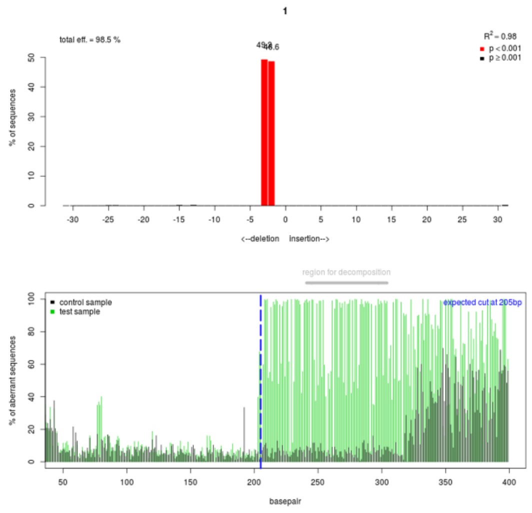

- Allele 1: incurred a 6bp deletion and a 3bp insertion across the DSB.
- Allele 2 incurred a 2bp deletion downstream from DSB

	Clone 1
**Allele 1**	CAGACACCATGGTGCACCTGACTCCTGAGGAG---TCTGCCGTTACTGCCCTGTGGGG
**WT**	CAGACACCATGGTGCACCTGACTCCTGAGGAGAAGTCTGCCGTTACTGCCCTGTGGGG
**Allele 2**	CAGACACCATGGTGCACCTGACTCCTGG--AAAAGTCTGCCGTTACTGCCCTGTGGGG

Site of interest
Insertions
Base changes

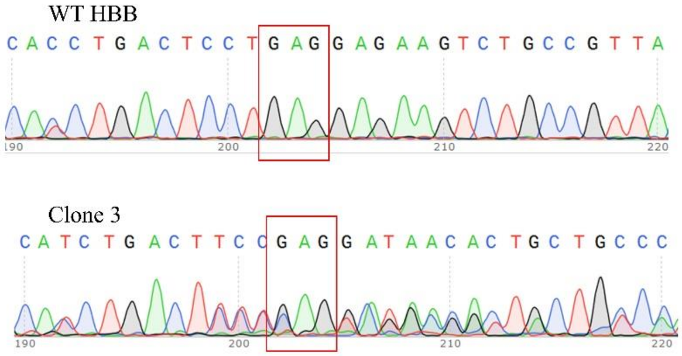

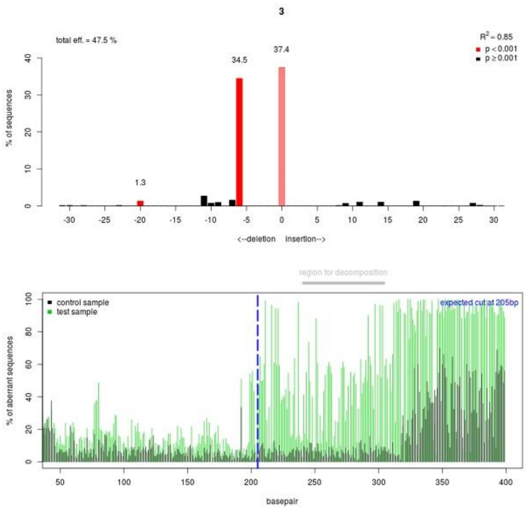

- Allele 1 had no insertions or deletions.
- Allele 2 incurred a 6bp deletion across the DSB.

	Clone 3
**Allele 1**	CAGACACCATGGTGCATCTGACTTTCCAGGAGAAGACTGCTGTCAATGCCCTGTGGGG
**WT**	CAGACACCATGGTGCACCTGACTCCTGAGGAGAAGTCTGCCGTTACTGCCCTGTGGGG
**Allele 2**	CAGACACCATGGTGCACCTGACTCCT------AAGTCTGCCGTTACTGCCCTGTGGGG

Site of interest
Insertions
Base changes

- Allele 1 incurred a 3bp deletion across the DSB
- Allele 2 and 3 incurred a 1bp deletion across the DSB

	Clone 4, 13, 21
Allele 1	CAGACACCATGGTGCATCTGACTCCTGWG---AAGTCTGCCGTTACTGCCCTGTGGGG
WT	CAGACACCATGGTGCACCTGACTCCTGAGGAGAAGTCTGCCGTTACTGCCCTGTGGGG
Allele 2	CAGACACCATGGTGCACCTGACTCCTGWG-AGAAGTCTGCCGTTACTGCCCTGTGGGG
Allele 3	CAGACACCATGGTGCACCTGACTCCTGWG-AGAAGTCTGCCGTTACTGCCCTGTGGGG

Site of interest
Insertions
Base changes

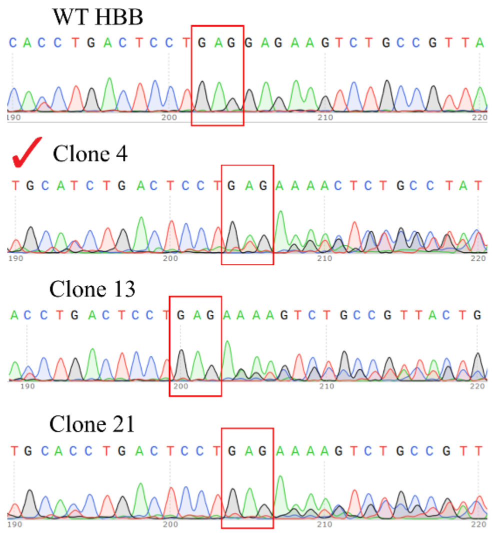

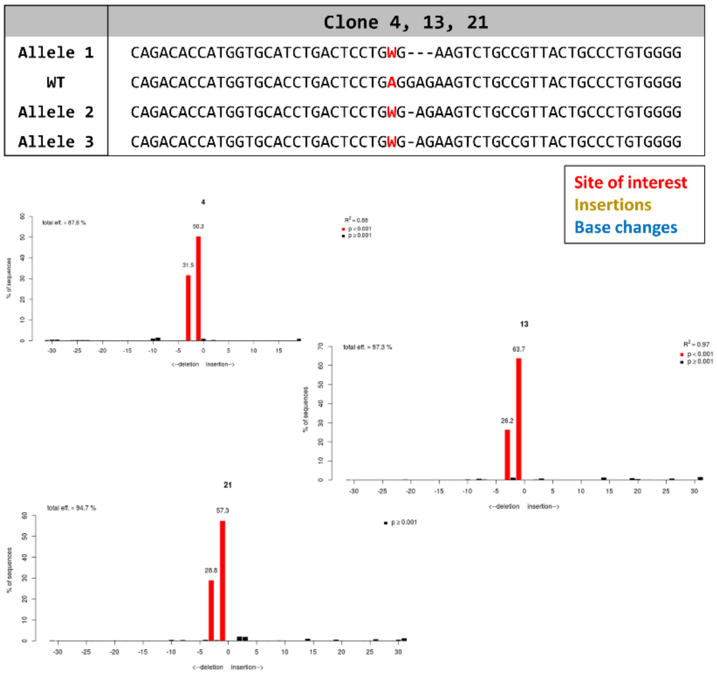

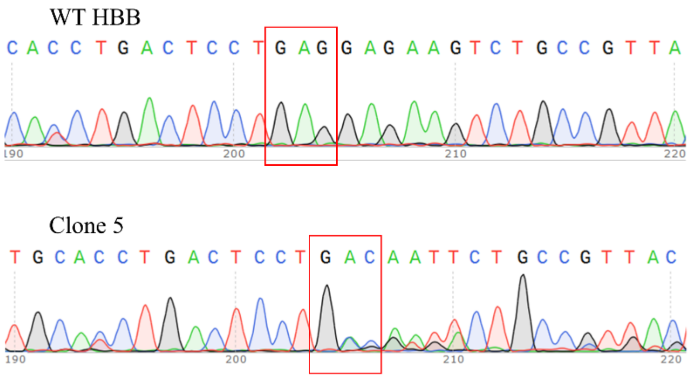

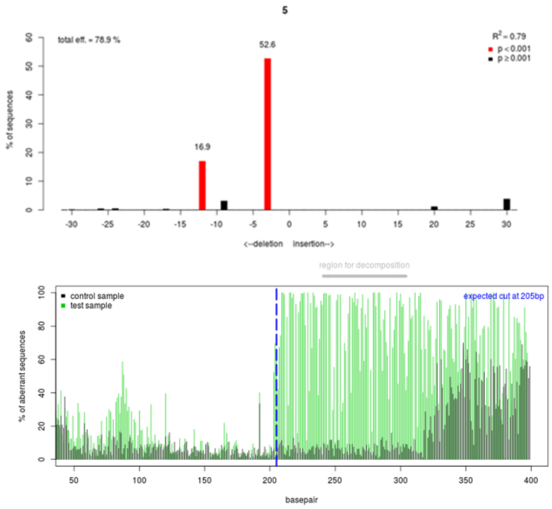

- Allele 1 incurred a 3bp deletion across the DSB
- Allele 2 and 3 incurred a 12bp deletion across the DSB

	Clone 5
Allele 1	CAGACACCATGGTGCATCTGACTCCTGAG---AAGTCTGCCGTTACTGCCCTGTGGGG
WT	CAGACACCATGGTGCACCTGACTCCTGAGGAGAAGTCTGCCGTTACTGCCCTGTGGGG
Allele 2	CAGACACCATGGTGCACCTGACTCCTG------------CCGTTACTGCCCTGTGGGG
Allele 3	CAGACACCATGGTGCACCTGACTCCTG------------CCGTTACTGCCCTGTGGGG

Site of interest
Insertions
Base changes

- Allele 1 and 2 incurred a 1bp deletion across the DSB site.

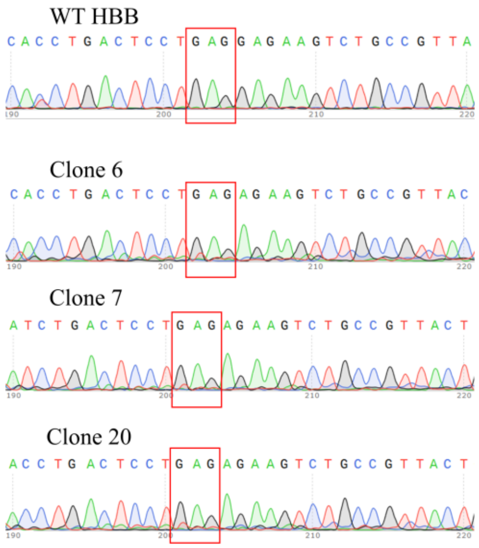

	Clone 6, 7, 20
Allele 1	CAGACACCATGGTGCACCTGACTCCTGAG-AGAAGTCTGCCGTTACTGCCCTGTGGGG
WT	CAGACACCATGGTGCACCTGACTCCTGAGGAGAAGTCTGCCGTTACTGCCCTGTGGGG
Allele 2	CAGACACCATGGTGCACCTGACTCCTGAG-AGAAGTCTGCCGTTACTGCCCTGTGGGG

Site of interest
Insertions
Base changes

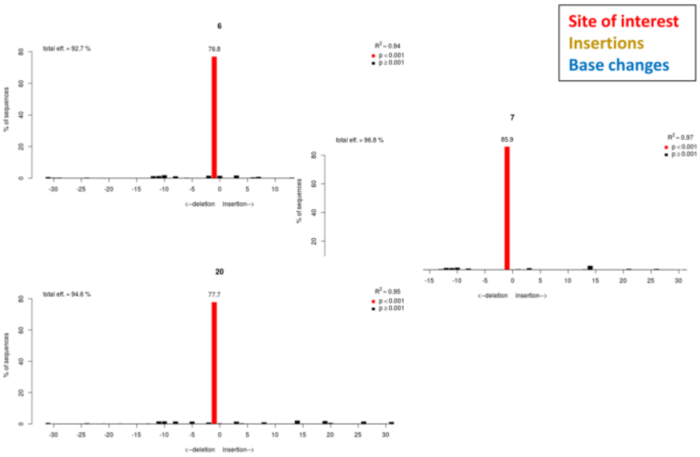

- Allele 1 and 2 incurred a 3bp deletion across the DSB site.

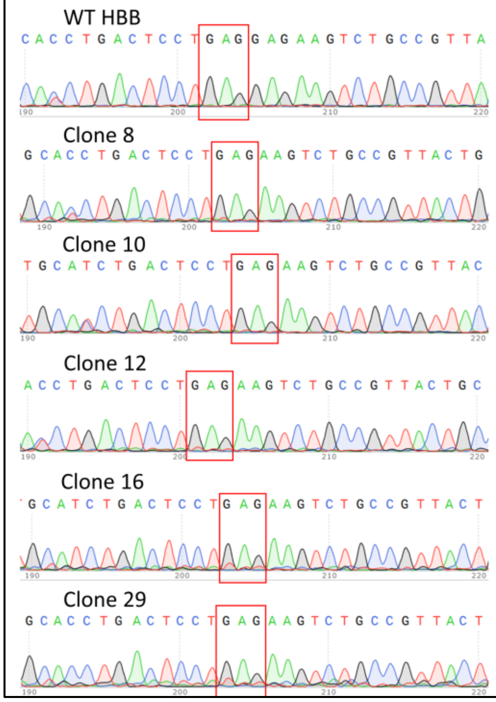

	Clone 8, 10, 12, 16, 29
Allele 1	CAGACACCATGGTGCACCTGACTCCTGAG---AAGTCTGCCGTTACTGCCCTGTGGGG
WT	CAGACACCATGGTGCACCTGACTCCTGAGGAGAAGTCTGCCGTTACTGCCCTGTGGGG
Allele 2	CAGACACCATGGTGCATCTGACTCCTGAG---AAGTCTGCCGTTACTGCCCTGTGGGG

Site of interest
Insertions
Base changes

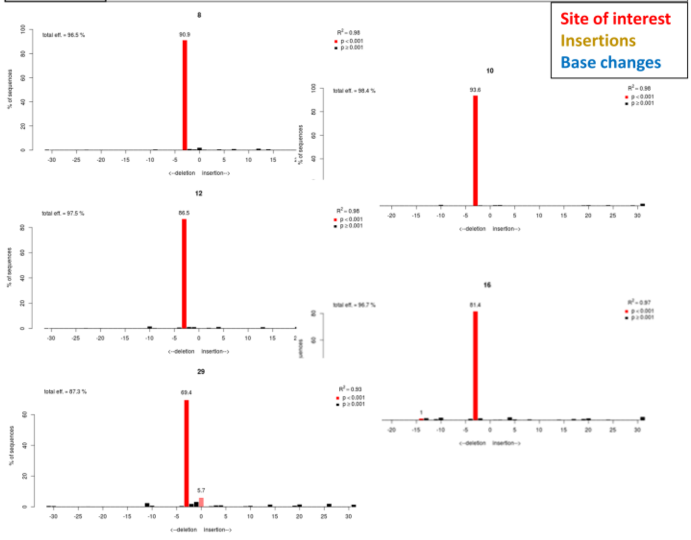

	Clone 9 & 11
Allele 1	CAGACACCATGGTGCACCTGACTCCTGAG---AAGTCTGCCGTTACTGCCCTGTGGGG
WT	CAGACACCATGGTGCACCTGACTCCTGAGGAGAAGTCTGCCGTTACTGCCCTGTGGGG
Allele 2	CAGACACCATGGTGCATCTGACTCCTGAG-AGAAGTCTGCCGTTACTGCCCTGTGGGG
*Allele 3 (9)	CAGACACCATGGTGCATCTGACTCCTGAG--**13bp del**---TTACTGCCCTGTGGGG
*Allele 3 (11)	CAGACACCATGGTGCATCTGACTC---------**40bp deletion**-----------

- Allele 1 incurred a 3bp deletion across the DSB site
- Allele 2 incurred a 1bp deletion across the DSB site
- Allele 3 incurred a 13bp deletion across the DSB site

Site of interest
Insertions
Base changes

WT HBB

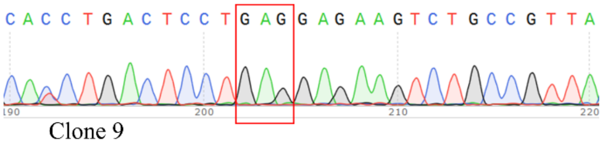

Clone 9

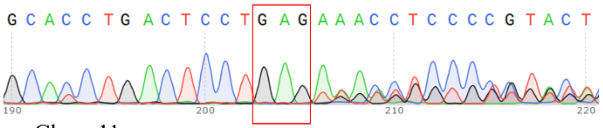

Clone 11

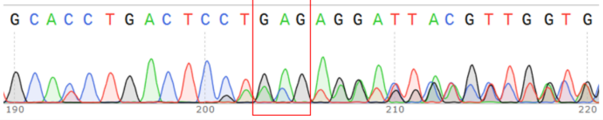

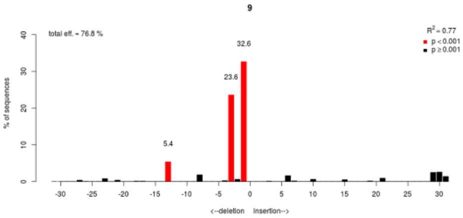

- Allele 1 incurred a 3bp deletion at the DSB site
- Allele 2 incurred a 1bp deletion at the DSB site
- Allele 3 incurred a 40bp across the DSB site

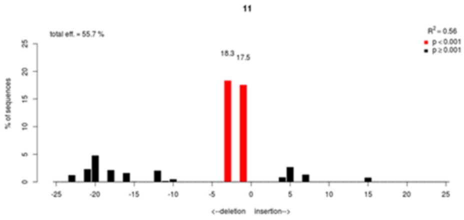

WT HBB

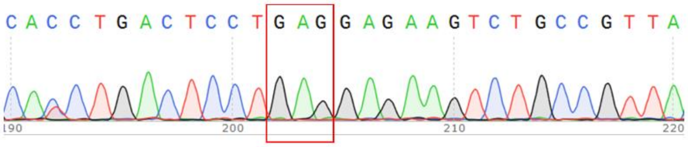

Clone 14

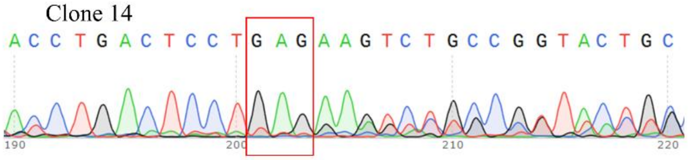

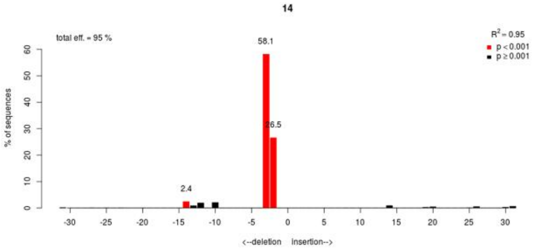

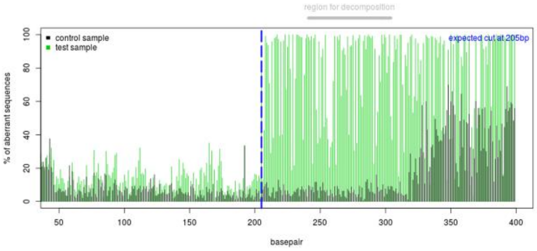

- Allele 1 incurred a 2bp deletion across the DSB
- Allele 2 and 3 a 3bp deletion across the DSB

	Clone 14
Allele 1	CAGACACCATGGTGCACCTGACTCCTGAG--GAAGTCTGCCGTTACTGCCCTGTGGGG
WT	CAGACACCATGGTGCACCTGACTCCTGAGGAGAAGTCTGCCGTTACTGCCCTGTGGGG
Allele 2	CAGACACCATGGTGCATCTGACTCCTGAG---AAGTCTGCCGTTACTGCCCTGTGGGG
Allele 3	CAGACACCATGGTGCATCTGACTCCTGAG---AAGTCTGCCGTTACTGCCCTGTGGGG

Site of interest
Insertions
Base changes

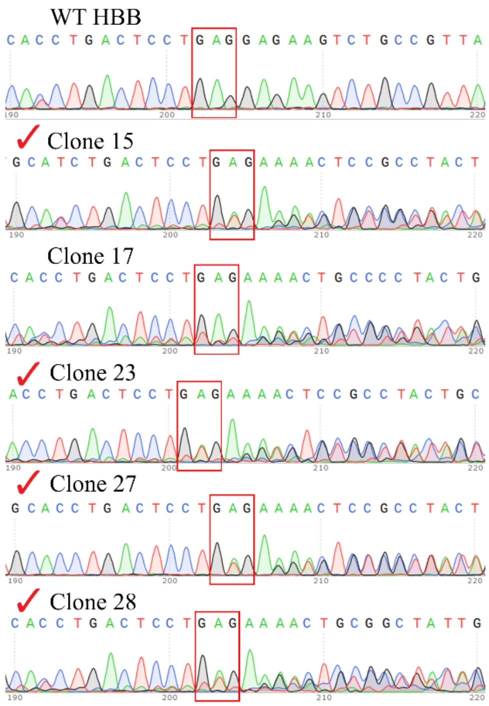

- Allele 1 incurred a 3bp deletion at the DSB
- Allele 2 incurred a 1bp deletion at the DSB

	Clone 15, 17, 23, 27, 28
**Allele 1**	CAGACACCATGGTGCACCTGACTCCTGWG---AAGTCTGCCGTTACTGCCCTGTGGGG
**WT**	CAGACACCATGGTGCACCTGACTCCTGAGGAGAAGTCTGCCGTTACTGCCCTGTGGGG
**Allele 2**	CAGACACCATGGTGCATCTGACTCCTGWG-AGAAGTCTGCCGTTACTGCCCTGTGGGG

Site of interest
Insertions
Base changes

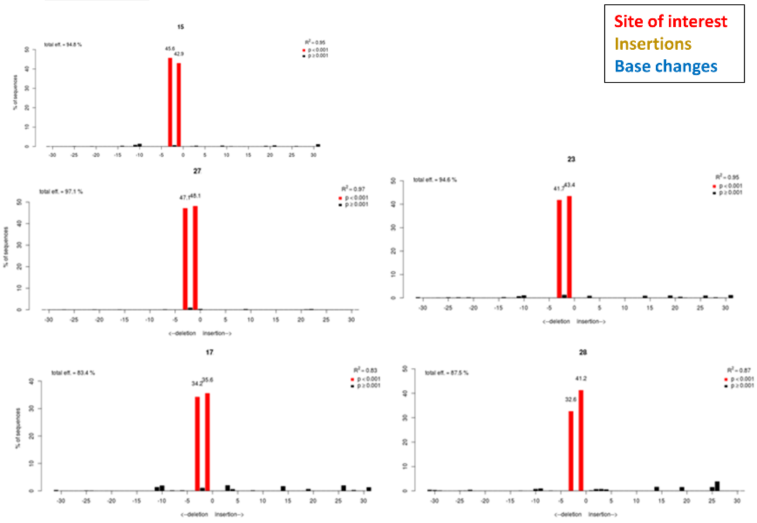

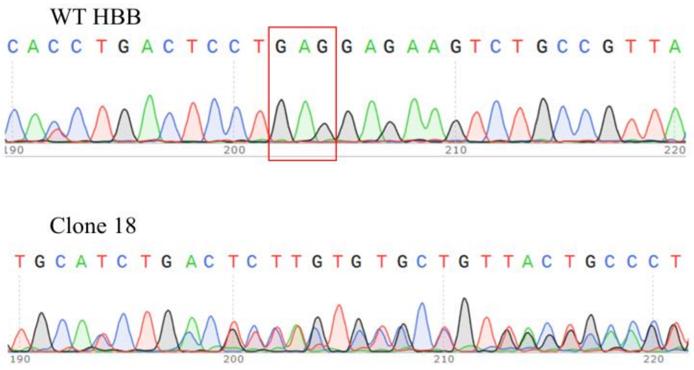

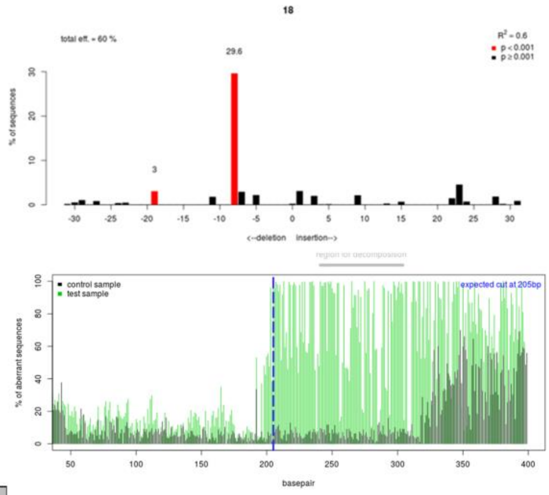

- Allele 1 incurred an 8bp upstream and downstream of DSB
- Allele 2 incurred a 19bp upstream and downstream of DSB

	Clone 18
**Allele 1**	CAGACACCATGGTGCACCTGACTCC--------AGTCTGCCGTTACTGCCCTGTGGGG
**WT**	CAGACACCATGGTGCACCTGACTCCTGAGGAGAAGTCTGCCGTTACTGCCCTGTGGGG
**Allele 2**	CAGACACCATGGTGCATCTG-------------------CCGTTACTGCCCTGTGGGG

Site of interest
Insertions
Base changes

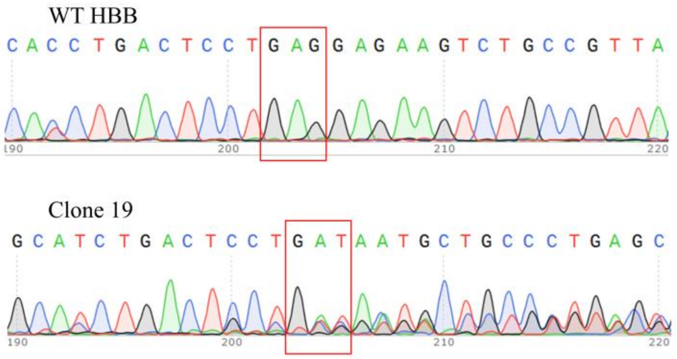

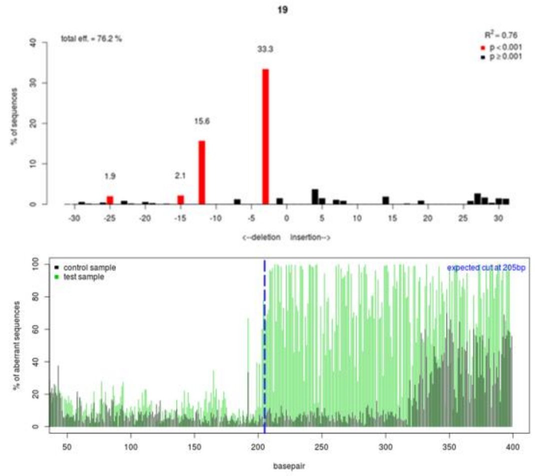

- Allele 1 incurred a 15bp deletion across the DSB site
- Allele 2 incurred a 3bp deletion across the DSB site
- Allele 3 incurred a 12bp deletion across the DSB site

	Clone 19
Allele 1	CAGACACCATGGTGCACCTGACT---**15bp del**----GCCGTTACTGCCCTGTGGGG
WT	CAGACACCATGGTGCACCTGACTCCTG**A**GGAGAAGTCTGCCGTTACTGCCCTGTGGGG
Allele 2	CAGACACCATGGTGCATCTGACTCCTG---AGAAGTCTGCCGTTACTGCCCTGTGGGG
Allele 3	CAGACACCATGGTGCATCTGACTCCTG--**12bp del**--CCGTTACTGCCCTGTGGGG

Site of interest
Insertions
Base changes

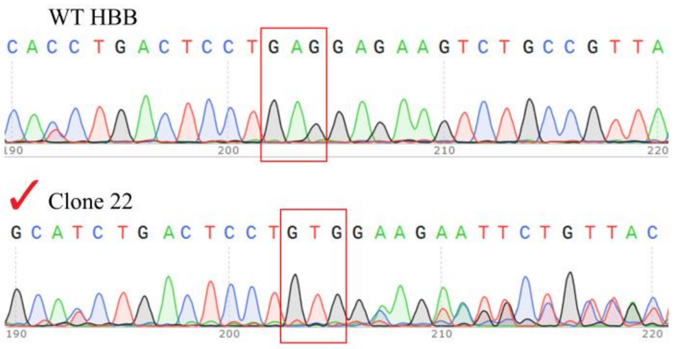

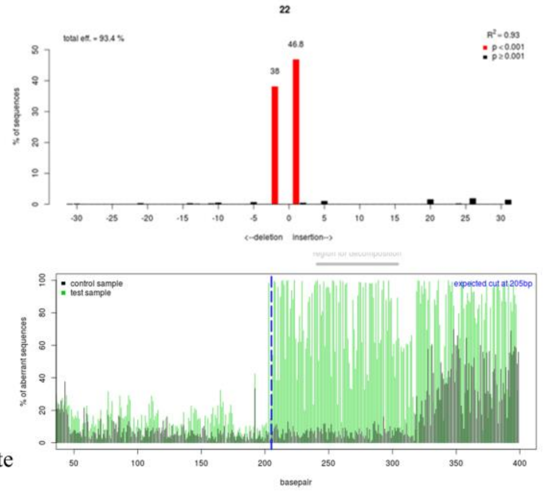

- Allele 1 incurred a 2bp deletion across the DSB site
- Allele 2 incurred a 1bp deletion with 2bp insertion across the DSB site

	Clone 22
Allele 1	CAGACACCATGGTGCATCTGACTCCTG**T**G--GAAGTCTGCCGTTACTGCCCTGTGGGG
WT	CAGACACCATGGTGCACCTGACTCCTG**A**GGAGAAGTCTGCCGTTACTGCCCTGTGGGG
Allele 2	CAGACACCATGGTGCACCTGACTCCTG**T**GGCAGAAGTCTGCCGTTACTGCCCTGTGGGG

Site of interest
Insertions
Base changes

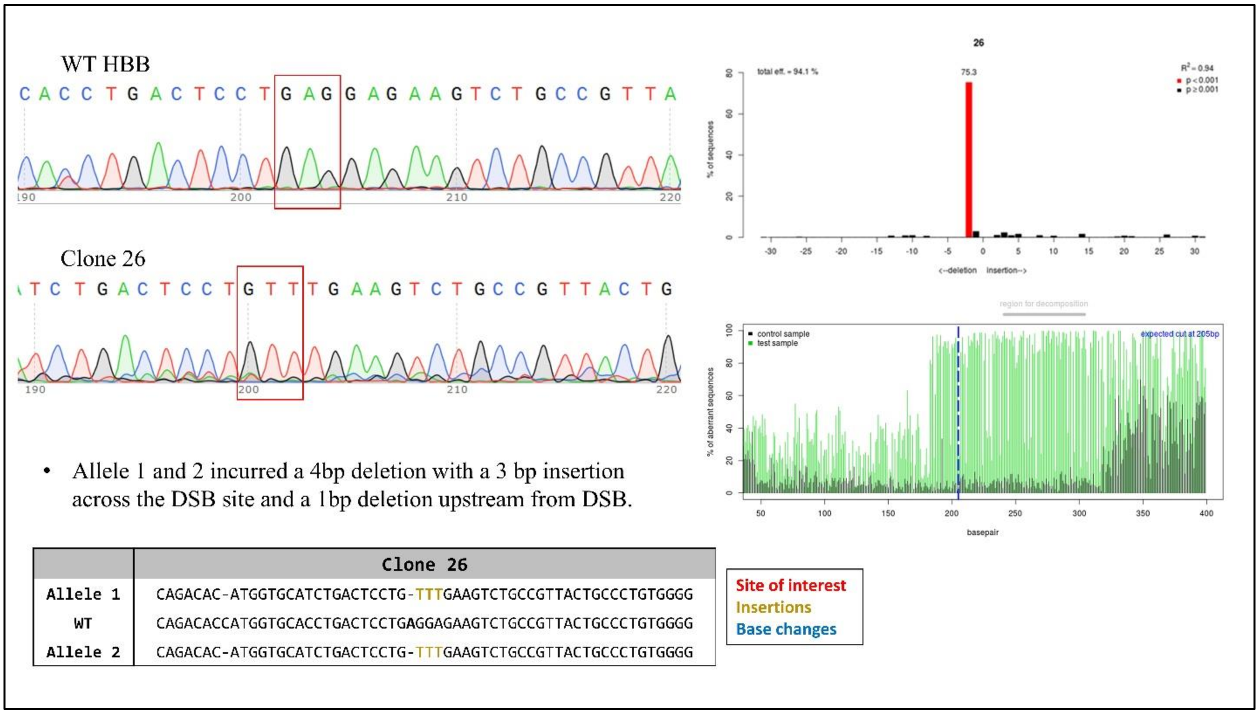

Supplementary Figure S2. Allelic Analysis of HBB in Clonally Expanded, Gene Edited K562 Cells.

Clone 1. The Sanger sequence and TIDE profile of Clone 1 displayed indel formations on each alleles. One allele incurred a six base pair deletion accompanied by a three base pair insertion, while the second allele incurred a two base pair deletion at the double stranded break site.

Clone 3. The Sanger sequence and TIDE profile of Clone 3 displayed indel formation on each allele. One allele incurred a six base pair deletion at the double stranded break site. The second allele displayed a correction pattern unlike those seen in any other clonally expanded population. In addition to a 4bp insertion proximal to the DSB site, the sequence of the allele in proximity bears exact homology to the analogous sequence of hemoglobin delta (HBD). This sequence homology exists only proximal to the DSB site, as sequences both upstream and further downstream of the DSB site exhibit exact homology to HBB.

Clones 4, 13, and 21. The Sanger sequences and TIDE profiles of Clones 4, 13, and 21 revealed that these three clones presented with the same sequence alterations around the target site, aside from the single nucleotide A to T conversion seen on one allele of Clone 4. One allele incurred a three base pair deletion while the second and third alleles both incurred a one base pair deletion at the double stranded break site.

Clone 5. The Sanger sequence and TIDE profile of Clone 5 displayed indel formation on both alleles. One allele incurred a three base pair deletion, while the second and third alleles incurred a 12 base pair deletion across the double stranded break site.

Clones 6, 7, and 20. The Sanger sequences and TIDE profiles of Clones 6, 7, and 20 revealed that these three clones presented the same sequence alterations around the target. Both alleles incurred a one base pair deletion at the double stranded break site.

Clones 8, 10, 12, 16, and 29. The Sanger sequences and TIDE profiles of Clones 8, 10, 12, 16, and 29 revealed that these five clones presented the same sequence alterations around the target. Both alleles incurred a three base pair deletion at the double stranded break site.

Clones 9 and 11. The Sanger sequences and TIDE profiles of Clones 9 and 11 revealed that both of these clones presented with the same sequence alterations around the target site, aside from the size of the large deletion seen on one of the alleles in both clones. One allele incurred a three base pair deletion, while the second allele incurred a one base pair deletion at the double stranded break site. The variation between these two clones is seen on the third allele, where in Clone 9 a 13 base pair deletion occurs and in Clone 11 a larger 40 base pair deletion occurs across the double stranded break site.

Clone 14. The Sanger sequence and TIDE profile of Clone 14 displayed indel formations on both alleles. One allele incurred a two base pair deletion, while the second and third allele incurred a three base pair deletion at the double stranded break site.

Clones 15, 17, 23, 27, and 28. The Sanger sequences and TIDE profiles of Clones 15, 17, 23, 27, and 28 revealed that these five clones presented the same sequence alterations around the target site; one allele incurred a 3 base pair deletion, the other allele incurred a 1 base pair deletion, and an A to T conversion on one allele with the exception of clone 17.

Clone 18. The Sanger sequence and TIDE profile of Clone 18 displayed indel formations on both alleles. One allele incurred an eight base pair deletion, while the second allele incurred a 19 base pair deletion across the double stranded break site.

Clone 19. The Sanger sequence and TIDE profile of Clone 19 displayed indel formations on both alleles. One allele incurred a 15 base pair, the second allele incurred a three base pair deletion, and the third allele incurred a 12 base pair deletion across the double stranded break site.

Clone 22. The Sanger sequence and TIDE profile of Clone 22 displayed indel formations and conversion of the single nucleotide A to T on both alleles. One of the alleles incurred a two base pair deletion, while the second allele incurred a one base pair insertion.

Clone 26. The Sanger sequence and TIDE profile of Clone 26 displayed indel formations on both alleles. Both alleles incurred a four base pair deletion accompanied by a three base pair insertion at the double stranded break site, with an additional single base pair deletion upstream from the double stranded break site.

Mutant eGFP targeting gRNA - AGCACTGCACGCCCTAGGTCAGG				
Top 10 potential genome-wide off-target sites				
**Sequence**	**Score**	**Mismatches**	**UCSC gene**	**Locus**
**AGCACTGCCCGCCCTAGGCCAGG**	1.7	2MMs [9:19]	NM_001199642	chr3:+123071187
**AGCTCTGCAGGCCCTAGGTGGAG**	1.3	3MMs [4:10:20]		chr11:-795678
**AGAGCTGCCTGCCCTAGGTCTAG**	0.8	4MMs [3:4:9:10]		chr1:-6697733
**AGCTCTGCACTACCTAGGTCAAG**	0.7	3MMs [4:11:12]		chr5:+141485541
**AACAGTGCATGCCCTAGGTACAG**	0.6	4MMs [2:5:10:20]		chr1:+89214834
**TGCACTGCAAGCCCTCGGTCAAG**	0.5	3MMs [1:10:16]	NM_030576	chr17:-61773589
**AGAAATGCCCTCCCTAGGTCCAG**	0.5	4MMs [3:5:9:11]	NM_207404	chr3:+42958472
**AGCCCTGGATGCCCTAGGCCAAG**	0.5	4MMs [4:8:10:19]		chr4:-4374838
**AGCCCTGCACGCCCTAGGGAAAG**	0.4	3MMs [4:19:20]	NM_139027	chr9:-136323253
**AGCCCTGCCTGCCCTAGGTGGAG**	0.4	4MMs [4:9:10:20]		chr9:-137677669

Supplementary Table S1. Top 10 Potential Predicted Off-target sites of the mutant eGFP Targeting gRNA in the Human Genome. The top 10 potential off-target sites are listed with the off-target sequence, off-target hit scores, the number of mismatches and their location in the seed sequence, the UCSC gene IDs (if off-target occurs in a gene), and the genomic locus of the off-target sites.
